# Supplementary material for: Chromatin regulators in the TBX1 network confer risk for conotruncal heart defects in 22q11.2DS
Source: NPJ Genom Med. 2023 Jul 18;8:17. doi: 10.1038/s41525-023-00363-y (PMC10354062; doi:10.1038/s41525-023-00363-y)
Supplement: Supplementary file 1 — Supplementary Figures [file 41525_2023_363_MOESM1_ESM.pdf]

Supplementary Fig. 1

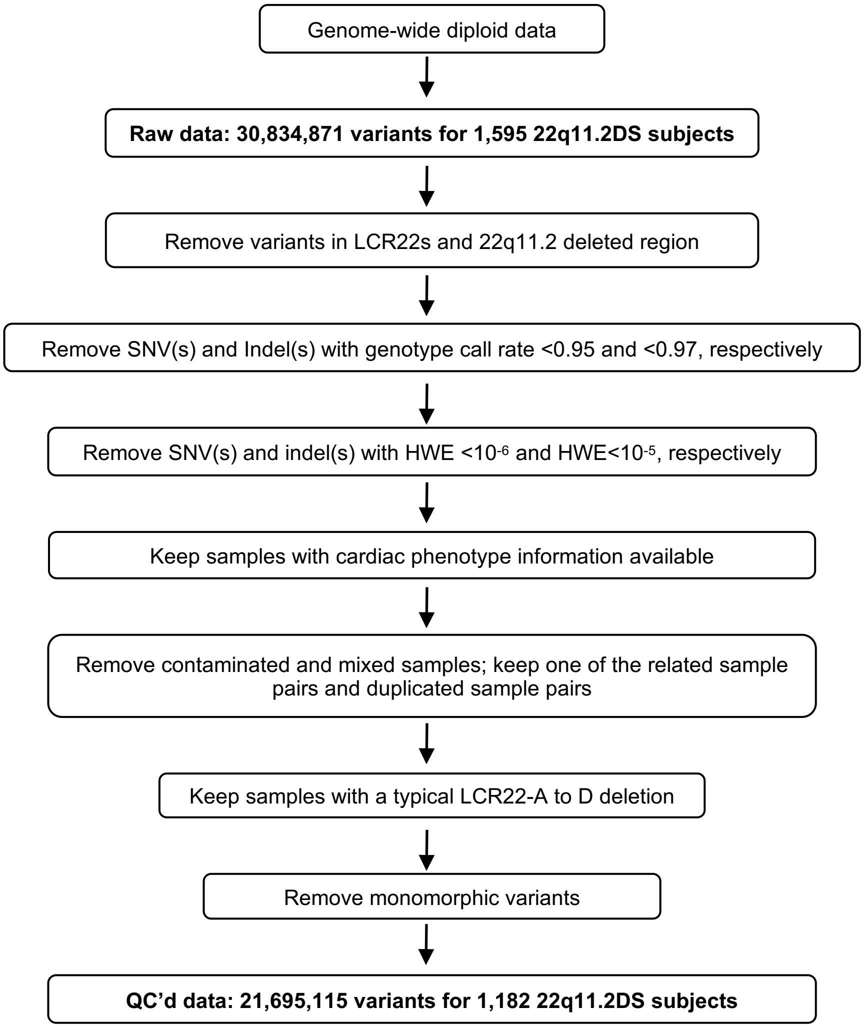

Supplementary Fig. 1. Detailed quality control (QC) procedures applied to unprocessed WGS data. Flow diagram of QC procedures used in this study. LCR22: Low Copy Repeats on chromosome 22q11.2; LCR22-A and LCR22-D mark two LCR22s that are 3 million base pairs (Mb) apart, in which flank the most typical deletion. HWE: Hardy-Weinberg equilibrium.

Supplementary Fig. 2

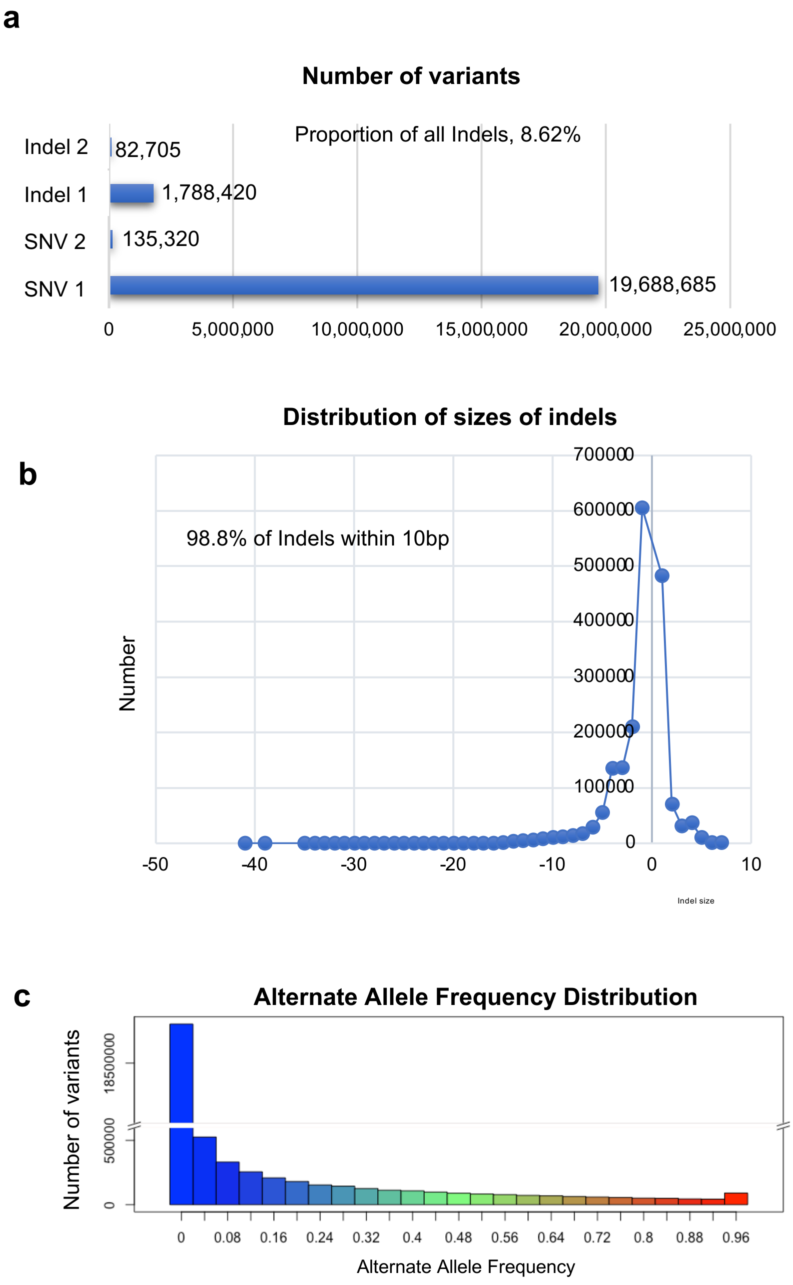

Supplementary Fig. 2. Summary of quality controlled WGS data. a, Bar plot shows the total number of the four variant categories, biallelic SNV (single nucleotide variant) and SNVs with more than one alternate allele (SNV2); biallelic indel and indels with more than one alternate allele (Indel 2). **b,** Distribution of number of indels based on size. A total of 98.8% of all indels are within 10bp. **c,** Histogram shows the distribution of the alternative allele frequency (AAF) and the vast majority are rare variants (AAF<0.01).

Supplementary Fig. 3

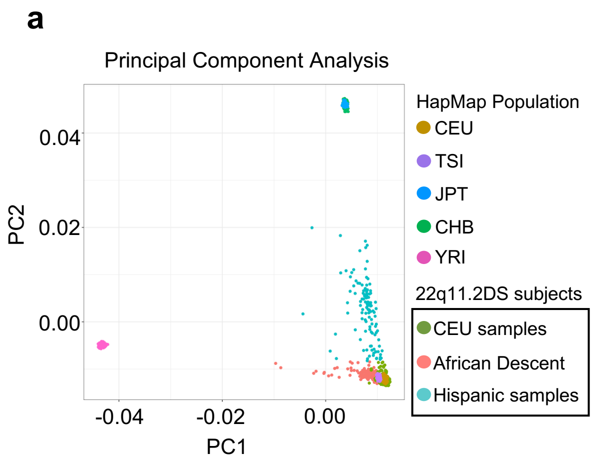

22q11.2DS on PCA	Subjects (%)	Subjects by cases and controls
Caucasian	879 (74.4%)	477 402
African Descent	184 (15.6%)	108 76
Hispanic	119 (10.0%)	59 60

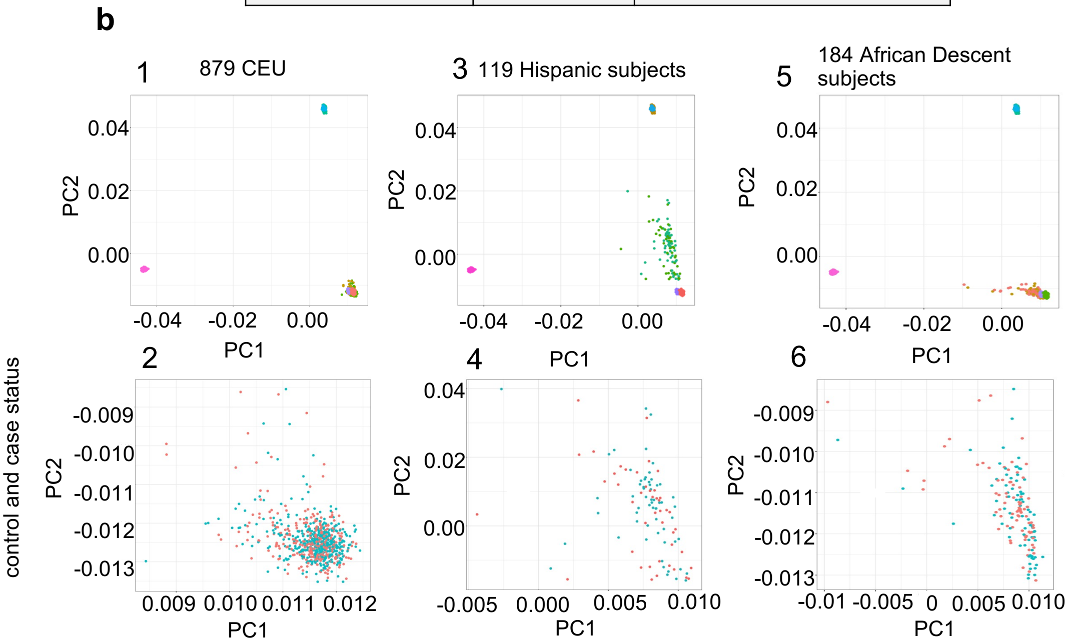

Supplementary Fig. 3. CTD cases and controls are matched for each ancestry group as determined by principal component analysis (PCA). a, PCA of 1,182 subjects to identify composition of race and ancestry. Reference population data is from HapMap Phase III and the 22q11.2DS cohort (22q11.2DS) data is indicated within CEU (Caucasian), Hispanic and AD (African descent) subjects based on the top PCs (black box). Table illustrates numbers. **b**, PCA within each of the three ancestry groups: **b1,** Scatter plot of 879 CEU samples by case control status (gold and green) against the same sets of HapMap samples plotted in **a**, **b2,** Zoomed in scatter plot of 879 CEU samples by case and control status (red and turquoise). **b3,** Scatter plot of 191 Hispanic samples by case control status (light green and green) against the same sets of HapMap samples plotted in Fig. 1b, **b4,** Zoomed in scatter plot of 191 Hispanic samples by case and control status (red and turquoise). **b5,** Scatter plot of 184 AD samples by case control status (light green and green) against the same set of HapMap samples plotted in Fig. 1b. **b6,** Zoomed in scatter plot of 184 AD samples by case and control status (red and turquoise). AD: African Descent; CEU: Utah residents with ancestry from Northern and Western Europe; TSI: Tuscan in Italy; YRI: Yoruba in Ibadan, Nigeria (West Africa); CHB: Han Chinese in Beijing, China; JPT: Japanese in Tokyo, Japan.

Supplementary Fig. 4

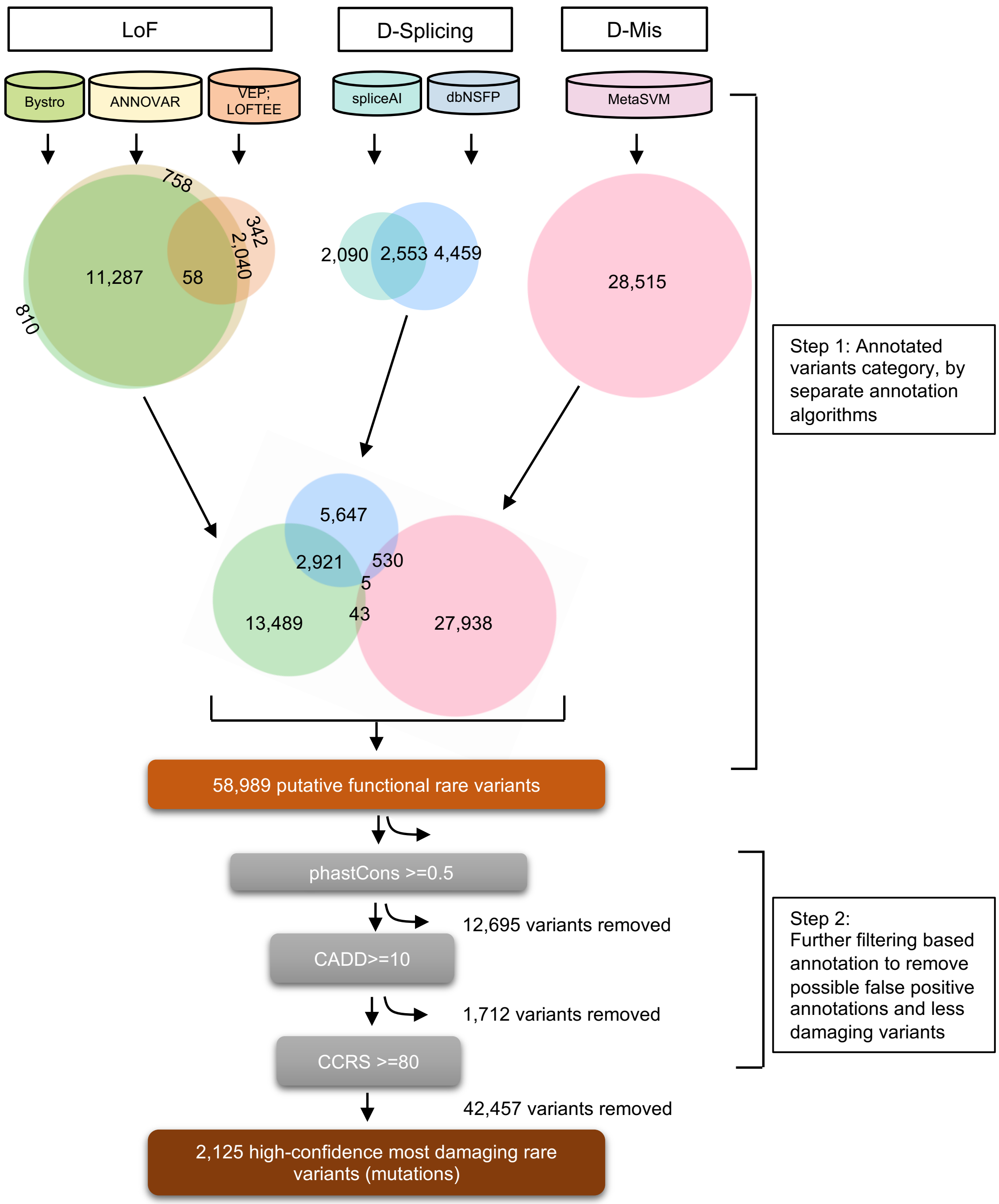

Supplementary Fig. 4. Identification of 2,125 MDRVs in the cohort from WGS. Bystro, ANNOVAR and VEP were used to annotate loss of function (LoF) variants in parallel (see Methods). MetaSVM was used to annotate damaging missense variants (D-Mis). Damaging splicing variants (D-splicing) were annotated by dbNSFP as well as spliceAI. All annotated variants were combined, totaling 58,989 rare putative functional variants and were further filtered by phastCons score >=0.5, CADD >=0.5 and CCRS >=80 sequentially to remove false positive variants. Finally, 2,125 high confidence MDRVs were identified. This was further narrowed to 1,861 variants when cross-compared with variants called by the GATK pipeline. LoF variants and D-Splicing variants are not mutually exclusive, and subset of the predicted canonical variants were also LoF. Synonymous variants as annotated by ANNOVAR and Bystro were used as negative controls for analyses. CCRS: Constrained coding regions.

Supplementary Fig. 5

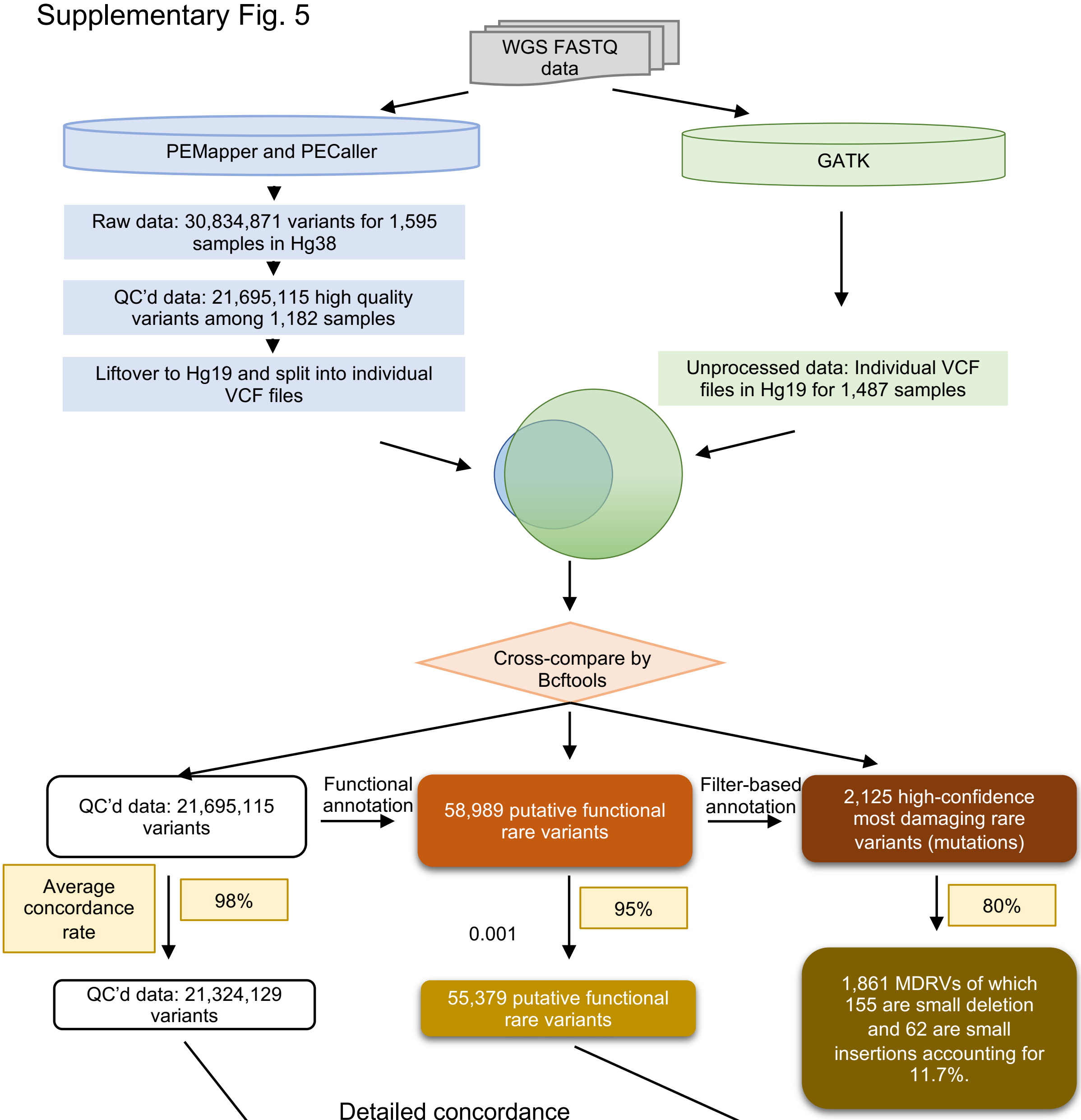

Detailed concordance rate

Concordance rate	All	Indel	SNVs
Consider GT	0.982±0.004	0.833±0.008	0.990±0.004
Not consider GT	0.982±0.001	0.841±0.007	0.990±0.001

Annotated variants	Number of variants	Concordance rate
Rare predicted functional variants	SNVs, 48,470	96.0%
	Indels, 10,519	90.6%
	Total, 58,989	95%

Supplementary Fig. 5. Flowchart and results of cross-comparison of variants identified by PEMapper/PECaller within the GATK pipeline and identification of 1,861 high confidence MDRVs by both pipelines. Of the 21,695,115 quality-controlled high-quality variants, 32,291 failed to liftover to hg19, comprising 0.15% of the total that were cross-compared. From the 2,125 variants identified using PEMapper/PECaller, 1,861 remained in GATK. VCF, variant call file.

Supplementary Fig. 6

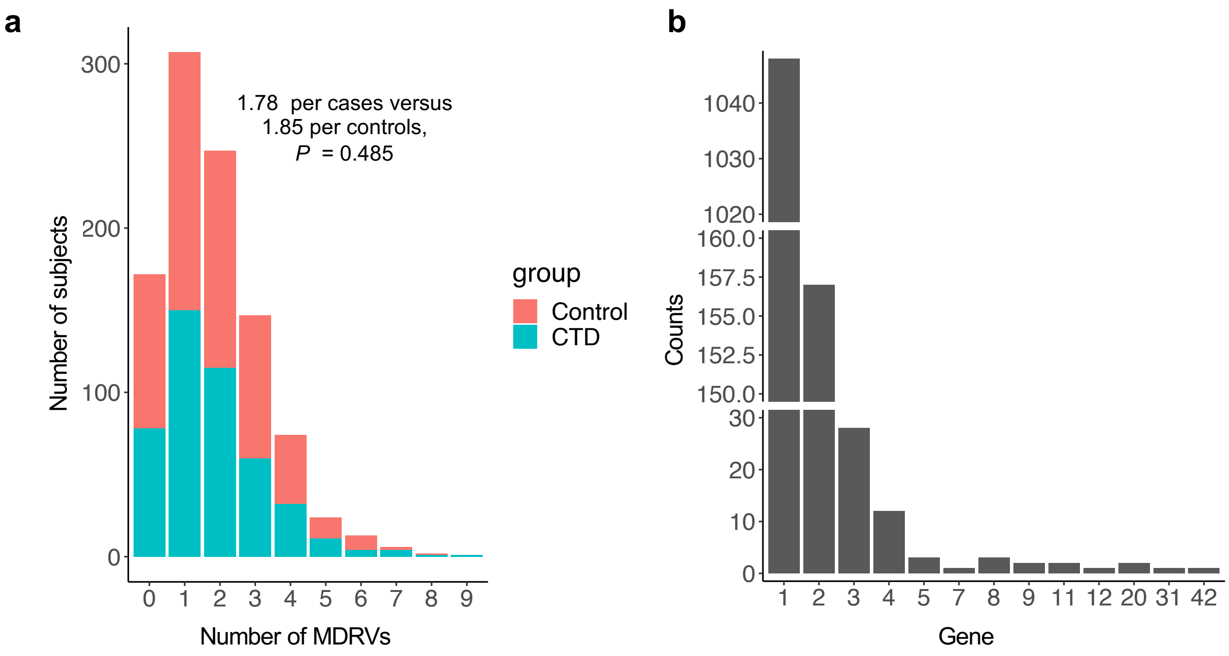

Supplementary Fig. 6. Distribution of the identified 1,861 MDRVs by case and control status and by gene. a, Bar plot of distribution of the number of MDRVs (most damaging rare variants) per case and control subject in the 22q11.2DS cohort**. b,** Bar plot of the distribution of the number of MDRVs per gene.

Supplementary Fig. 7a-c

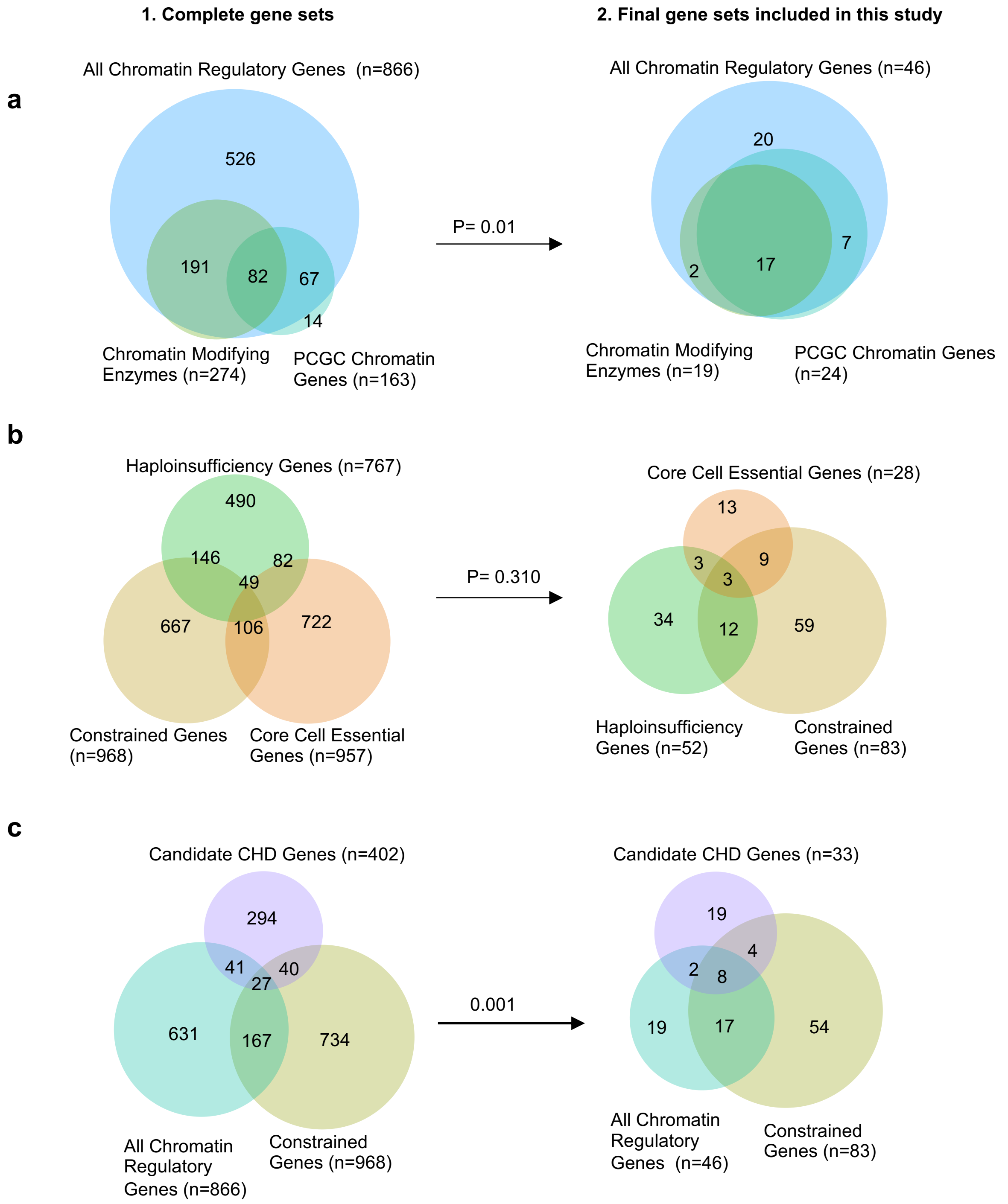

Supplementary Fig. 7. Overlap of genes within gene sets as well as those of corresponding gene sets for the final number of genes included in the weighted gene set based test. a, Overlap of the All Chromatin Regulatory Genes set that was compiled from 81 gene set enrichment analysis (GSEA) terms (https://www.gsea-msigdb.org/gsea/index.jsp) (n=866, 46), one gene set was generated using the specific REACTOME term: Chromatin Modifying Enzymes (n=274,19), and the 163 Chromatin Genes set was curated by the PCGC (n=163, 24). **b,** Overlap of the Constrained Genes set (n=968, 83), Core Cell Essential Genes set (n=957, 28) and Haploinsufficiency Genes set (n=767,52) for the complete and final gene sets in the cohort. **c,** Overlap of the genes within the complete and final gene sets of Constrained Genes (n=968, 83), All Chromatin Regulatory Genes (n=866,46) and Candidate CHD Genes (n=402, 33), respectively.

Supplementary Fig. 7, D and E.

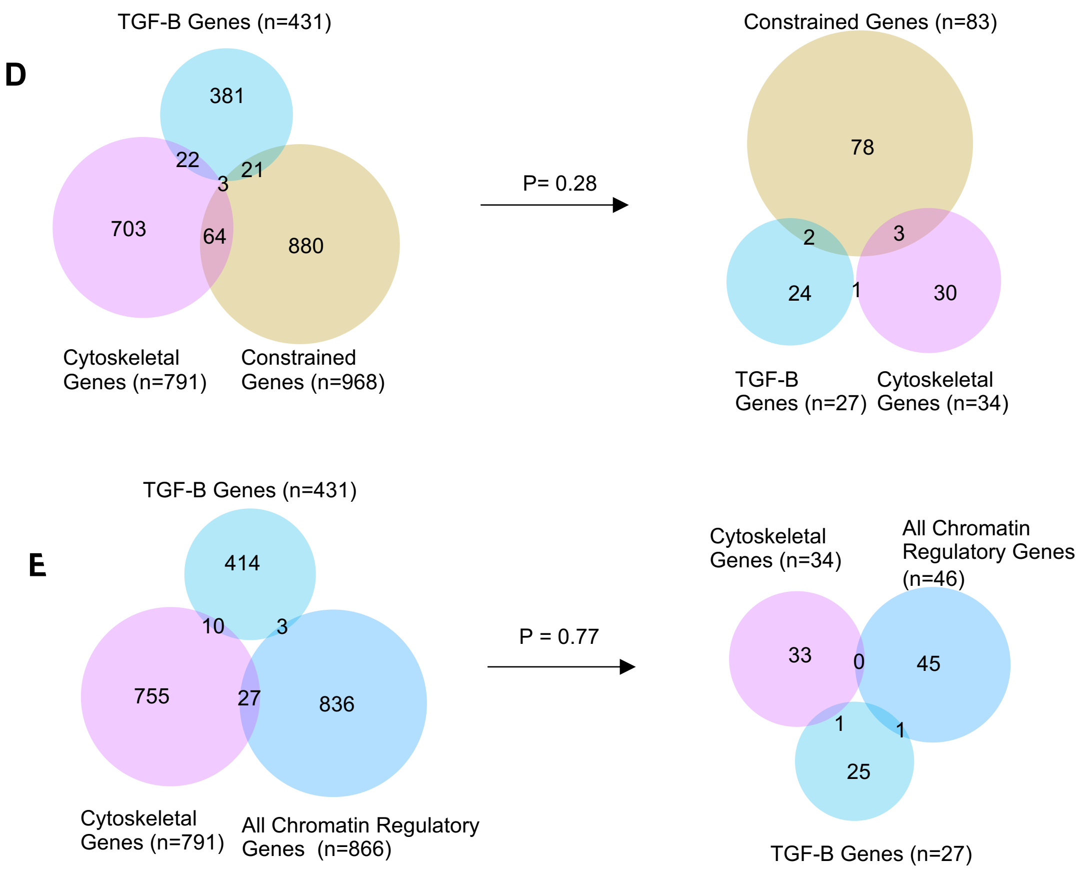

Supplementary Fig. 7. Overlap of genes within gene sets as well as those of corresponding gene sets for the final number of genes included in the weighted gene set based test. d, Overlap of gene sets of Constrained Genes (n=968, 83), TGF-B Genes (n=431, 27) and Cytoskeletal Genes (n=791, 34). **e,** Overlap of gene sets of Cytoskeletal Genes (n=791,34), All Compiled Chromatin Regulatory Genes (n=866, 46), and TGF-B Genes (n=431,27). *P* values from the proportion test for the enrichment of the shared genes are indicated.
